# Supplementary material for: Comparison of Single-Port Laparoscopy with Other Surgical Approaches in Endometrial Cancer Surgical Staging: Propensity-Score-Matched Analysis
Source: Cancers (Basel). 2023 Nov 8;15(22):5322. doi: 10.3390/cancers15225322 (PMC10670050; doi:10.3390/cancers15225322)
Supplement: Supplementary file 1 [file cancers-15-05322-s001.zip › cancers-2689334-supplementary.pdf]

cancers

MDPI

Comparison of Single-Port Laparoscopy with Other Surgical Approaches in Endometrial Cancer Surgical Staging: Propensity Score Matched Analysis

Sang Hyun Cho, Jung-Yun Lee, Eun Ji Nam, Sunghoon Kim, Young Tae Kim and Sang Wun Kim

Table S1. Patient characteristics of four different surgical staging methods for endometrial cancer (n=881).

	Total (n = 881)	SPL (n = 107)	MPL (n = 299)	RAL (n =2 07)	LT (n = 268)	p-value
Age, mean ± SD, years	54.1 ± 10.2	54.1 ± 11.1	53.8 ± 10.6	53.0 ± 9.2	55.3 ± 10.2	.113
Body mass index, mean ± SD, kg/m^2	24.8 ± 4.2	24.8 ± 3.6	24.8 ± 4.5	24.6 ± 4.4	24.8 ± 4.0	.66
Prior abdominal surgeries, n (%)						.367
No	498 (56.5)	66 (61.7)	174 (58.2)	117 (56.5))	141 (52.6)	
Yes	383 (43.5)	41 (38.3)	125 (41.8)	90 (43.5)	127 (47.4)	
FIGO stage, n (%)						<.001[1]
I	698 (79.2)	88 (82.2)	258 (86.3)	177 (85.5)	175 (65.3)	
IA	592 (67.2)	75 (70.1)	229 (76.6)	155 (74.9)	133 (49.6)	
IB	106 (12.0)	13 (12.1)	29 (9.7)	22 (10.6)	42 (15.7)	
II	55 (6.2)	5 (4.7)	14 (4.7)	9 (4.3)	27 (10.1)	
II	52 (5.9)	4 (3.7)	14 (4.7)	8 (3.9)	26 (9.7)	
IIA	0	0	0	0	0	
IIB	3 (0.3)	1 (0.9)	0	1 (0.5)	1 (0.4)	
III	103 (11.7)	14 (13.1)	24 (8.0)	19 (9.2)	46 (17.2)	
IIIA	19 (2.2)	1 (0.9)	7 (2.3)	6 (2.9)	5 (1.9)	
IIIB	7 (0.8)	0 (0)	4 (1.3)	0 (0)	3 (1.1)	
IIIC	2 (0.2)	0 (0)	0 (0)	0 (0)	2 (0.7)	
IIIC1	37 (4.2)	6 (5.6)	8 (2.7)	6 (2.9)	17 (6.3)	
IIIC2	38 (4.3)	7 (6.5)	5 (1.7)	7 (3.4)	19 (7.1)	
IV	25 (2.8)	0	3 (1.0)	2 (1.0)	20 (7.5)	
IVA	5 (0.6)	0 (0)	0 (0)	1 (0.5)	4 (1.5)	
IVB	20 (2.3)	0 (0)	3 (1.0)	1 (0.5)	16 (6.0)	
Histology, n (%)						<.001[2]
Endometrioid	732 (83.1)	90 (84.1)	271 (90.6)	184 (88.9)	187 (69.8)	
Non-endometrioid	149 (16.9)	17 (15.9)	28 (9.4)	23 (11.1)	81 (30.2)	
Serous	37 (4.2)	5 (4.7)	8 (2.7)	6 (2.9)	18 (6.7)	
Clear cell	14 (1.6)	0	2 (0.7)	2 (1.0)	10 (3.7)	
Carcinosarcoma	35 (4.0)	6 (5.6)	4 (1.3)	5 (2.4)	20 (7.5)	
Mucinous	5 (0.6)	0	3 (1.0)	1 (0.5)	1 (0.4)	
Mixed	24 (2.7)	0	5 (1.7)	5 (2.4)	14 (5.2)	
Neuroendocrine	3 (0.3)	1 (0.9)	0	1 (0.5)	1 (0.4)	
Mesonephric adenocarcinoma	5 (0.6)	1 (0.9)	2 (0.7)	1 (0.5)	1 (0.4)	
Adenosarcoma	4 (0.5)	0	0	1 (0.5)	3 (1.1)	
Leiomyosarcoma	3 (0.3)	0	1 (0.3)	0	2 (0.7)	
High-grade ESS	1 (0.1)	0	0	0	1 (0.4)	
Low-grade ESS	14 (1.6)	4 (3.7)	1 (0.3)	1 (0.5)	8 (3.0)	
STUMP	1 (0.1)	0	0	0	1 (0.4)	
Others	3 (0.3)	0	2 (0.7)	0	1 (0.4)	

FIGO differentiation grade, n (%)						<.001
Grade 1	423 (48.0)	59 (55.1)	167 (55.9)	105 (50.7)	92 (34.3)	
Grade 2	250 (28.4)	23 (21.5)	85 (28.4)	63 (30.4)	79 (29.5)	
Grade 3	153 (17.4)	15 (14.0)	30 (10.0)	34 (16.4)	74 (27.6)	
Not reported	55 (6.2)	10 (9.3)	17 (5.7)	5 (2.4)	23 (8.6)	
Myometrial invasion, n (%)						<.001
<50%	646 (73.3)	79 (73.8)	245 (81.9)	165 (79.7)	157 (58.6)	
≥50%	221 (25.1)	24 (22.4)	53 (17.7)	35 (16.9)	109 (40.7)	
Not reported	14 (1.6)	4 (3.7)	1 (0.3)	7 (3.4)	2 (0.7)	
Lymphovascular space invasion, n (%)						<.001
Yes	176 (20.0)	28 (26.2)	39 (13.0)	38 (18.4)	71 (26.5)	
No	705 (80.0)	79 (73.8)	260 (87.0)	169 (81.6)	197 (73.5)	
Cervical stromal invasion, n (%)						<.001
Yes	114 (12.9)	10 (9.3)	26 (8.7)	14 (6.8)	64 (23.9)	
No	766 (86.9)	97 (90.7)	272 (91.0)	193 (93.2)	204 (76.1)	
Not reported	1 (0.1)	0	1 (0.3)	0	0	
Parametrial invasion, n (%)						<.001
Yes	39 (4.4)	3 (2.8)	7 (2.3)	2 (1.0)	27 (10.1)	
No	838 (95.1)	104 (97.2)	290 (97.0)	205 (99.0)	239 (89.2)	
Not reported	4 (0.5)	0	2 (0.7)	0	2 (0.7)	
PLN dissection/sampling, n (%)	837 (95.0)	101 (94.4)	288 (96.3)	206 (99.5)	242 (90.3)	<.001
PALN dissection/sampling, n (%)	710 (80.6)	96 (89.7)	226 (75.6)	178 (86.0)	210 (78.4)	.002
PLN metastasis, n (%)	80 (9.1)	12 (11.2)	12 (4.0)	12 (5.8)	44 (16.4)	<.001
PALN metastasis, n (%)	46 (5.2)	7 (6.5)	6 (2.0)	7 (3.4)	26 (9.7)	<.001

Abbreviations: ESS, endometrial stromal sarcoma; FIGO, International Federation of Gynecology and Obstetrics; LT, laparotomy; MPL, multi-port laparoscopy; PALN, paraaortic lymph node; PLN, pelvic lymph node; SD, standard deviation; SPL, single-port laparoscopy; STUMP, uterine smooth muscle tumor of uncertain malignant potential; RAL, robot-assisted laparoscopy; [1]χ^2 test for FIGO stage I vs. II vs. III vs. IV; [2]χ^2 test for endometrioid vs. non-endometrioid tumors.

Table S2. Patient characteristics of four different surgical staging methods for endometrial cancer; after propensity score matching.

	SPL (n = 107)	MPL (n = 107)	RAL (n = 107)	LT (n = 107)	p-value
Age, mean ± SD, years	54.1 ± 11.1	54.2 ± 9.4	53.5 ± 9.5	53.9 ± 9.2	.775
Body mass index, mean ± SD, kg/m^2	24.8 ± 3.6	24.8 ± 4.3	24.7 ± 4.1	25.2 ± 3.7	.497
FIGO stage, n (%)					.923
IA	75 (70.1)	80 (74.8)	77 (72.0)	68 (63.6)	
IB	13 (12.1)	12 (11.2)	13 (12.1)	19 (17.8)	
II	4 (3.7)	3 (2.8)	3 (2.8)	2 (1.9)	
IIB	1 (0.9)	0	1 (0.9)	0	
IIIA	1 (0.9)	1 (0.9)	1 (0.9)	2 (1.9)	
IIIB	0	0	0	0	
IIIC1	6 (5.6)	6 (5.6)	5 (4.7)	4 (3.7)	
IIIC2	7 (6.5)	3 (2.8)	6 (5.6)	8 (7.5)	
IVA	0	0	0	1 (0.9)	
IVB	0	2 (1.9)	1 (0.9)	3 (2.8)	
Histology, n (%)					.119
Endometrioid	90 (84.1)	95 (88.8)	93 (86.9)	90 (84.1)	
Serous	5 (4.7)	4 (3.7)	4 (3.7)	3 (2.8)	

Clear cell	0	0	0	3 (2.8)	
Carcinosarcoma	6 (5.6)	2 (1.9)	2 (1.9)	5 (4.7)	
Mixed	0	2 (1.9)	4 (3.7)	3 (2.8)	
Neuroendocrine	1 (0.9)	0	1 (0.9)	0	
Mesonephric adenocarcinoma	1 (0.9)	1 (0.9)	1 (0.9)	0	
Adenosarcoma	0	0	0	1 (0.9)	
Leiomyosarcoma	0	1 (0.9)	0	0	
Low-grade ESS	4 (3.7)	0	1 (0.9)	0	
STUMP	0	0	0	1 (0.9)	
Others	0	2 (1.9)	0	1 (0.9)	
FIGO differentiation grade, n (%)					.157
Grade 1	59 (55.1)	59 (55.1)	55 (51.4)	59 (55.1)	
Grade 2	23 (21.5)	29 (27.1)	29 (27.1)	23 (21.5)	
Grade 3	15 (14.0)	9 (8.4)	21 (19.6)	19 (17.8)	
Not reported	10 (9.3)	10 (9.3)	2 (1.9)	6 (5.6)	
Myometrial invasion, n (%)					.068
<50%	79 (73.8)	85 (79.4)	80 (74.8)	69 (64.5)	
≥50%	24 (22.4)	22 (20.6)	23 (21.5)	36 (33.6)	
Not reported	4 (3.7)	0	4 (3.7)	2 (1.9)	
Lymphovascular space invasion, n (%)					.925
Yes	79 (73.8)	80 (74.8)	76 (71.0)	77 (72.0)	
No	28 (26.2)	27 (25.2)	31 (29.0)	30 (28.0)	
Cervical stromal invasion, n (%)					.955
Yes	97 (90.7)	98 (91.6)	99 (92.5)	97 (90.7)	
No	10 (9.3)	9 (8.4)	8 (7.5)	10 (9.3)	
Parametrial invasion, n (%)					.337
Yes	104 (97.2)	104 (97.2)	105 (98.1)	100 (93.5)	
No	3 (2.8)	3 (2.8)	2 (1.9)	7 (6.5)	
PLN dissection/sampling, n (%)	101 (94.4)	99 (92.5)	106 (99.1)	101 (94.4)	.147
PALN dissection/sampling, n (%)	96 (89.7)	93 (86.9)	103 (96.3)	97 (90.7)	.114
PLN metastasis, n (%)	12 (11.2)	10 (9.3)	11 (10.3)	14 (13.1)	.841
PALN metastasis, n (%)	7 (6.5)	4 (3.7)	6 (5.6)	9 (8.4)	.546

Abbreviations: FIGO, International Federation of Gynecology and Obstetrics; LT, laparotomy; MPL, multi-port laparoscopy; PALN, paraaortic lymph node; PLN, pelvic lymph node; RAL, robot-assisted laparoscopy; SD, standard deviation; SMD, standard mean deviation; SPL, single-port laparoscopy.

Table S3. Perioperative surgical outcomes and adjuvant treatments of four endometrial cancer surgical staging methods; after propensity score matching.

	SPL (n = 107)	MPL (n = 107)	RAL (n = 107)	LT (n = 107)	p-value
Operative time, mean ± SD, min	205.1 ± 76.9	167.2 ± 77.2	177.1 ± 56.5	163.4 ± 51.0	<.001
Estimated blood loss, mean ± SD, mL	69.5 ± 90.8	140.3 ± 157.6	120.2 ± 126.8	368.3 ± 326.4	<.001
Intraoperative transfusion, n (%)	0	2 (1.9)	1 (0.9)	4 (3.7)	.225
Postoperative hospital stay, mean ± SD, days	5.2 ± 2.3	7.4 ± 2.8	7.5 ± 2.2	10.3 ± 4.6	<.001
Harvested PLNs, mean ± SD, n	16.1 ± 9.5	17.9 ± 9.8	15.5 ± 8.0	21.2 ± 9.2	<.001

Harvested PALNs, mean ± SD, n	14.1 ± 10.7	7.3 ± 7.3	6.1 ± 6.3	5.2 ± 4.5	<.001
Adjuvant therapy, n (%)					
Radiotherapy	24 (22.4)	24 (22.4)	23 (21.5)	33 (31.1)	.315
Chemotherapy	32 (29.9)	17 (15.9)	22 (20.6)	20 (18.7)	.070
Hormonal therapy	3 (2.8)	0	0	0	.061
None	58 (54.2)	74 (69.2)	69 (64.5)	60 (56.1)	.081

Abbreviations: LT, laparotomy; MPL, multi-port laparoscopy; PALN, para-aortic lymph node; PLN, pelvic lymph node; RAL, robot-assisted laparoscopy; RT, radiotherapy; SD, standard deviation; SPL, single-port laparoscopy; VBT, vaginal brachytherapy.

Table S4. Complications of four endometrial cancer surgical staging methods; after propensity score matching.

**Complications***	**SPL (n = 107)**	**MPL (n = 107)**	**RAL (n = 107)**	**LT (n = 107)**	**p-value**
Intraoperative complications, n (%)					
Bowel injury	1 (0.9)	0	1 (0.9)	2 (1.9)	.905
Vessel injury	0	1 (0.9)	1 (0.9)	0	1.000
Vagina injury	1 (0.9)	0	0	0	1.000
Ureter injury	1 (0.9)	0	0	0	1.000
Bladder injury	0	0	2 (1.9)	0	.248
Nerve injury	0	1 (0.9)	0	0	1.000
Total	3 (2.8)	1 (0.9)	4 (3.7)	2 (1.9)	.712
Postoperative complications, n (%)					
Fever	0	1 (0.9)	0	0	1.000
Incisional hernia	0	0	0	0	-
Wound problems (dehiscence/infection/discharge)	0	0	0	6 (5.6)	.001
Hydronephrosis/Ureter stricture	0	0	0	1 (0.9)	1.000
Voiding difficulty	0	0	1 (0.9)	0	1.000
Vagina vault problems (discharge/bleeding/necrotic change/prolapse/vault dehiscence)	0	0	1 (0.9)	0	1.000
DVT/PTE	1 (0.9)	0	0	1 (0.9)	1.000
Cellulitis	0	0	0	0	-
Nerve injury	0	0	0	0	-
Peritonitis	1 (0.9)	0	0	0	1.000
Ileus/bowel obstruction	0	0	0	1 (0.9)	1.000
Hematoma	0	0	0	0	-
Hematuria	0	0	0	0	-
Infarction	0	0	0	0	-
Total	2 (1.9)	1 (0.9)	2 (1.9)	8 (7.5)	.052

*Each complication in a patient was counted individually. Abbreviations: DVT/PTE, deep vein thrombosis/pulmonary thromboembolism; LT, laparotomy; MPL, multi-port laparoscopy; RAL, robot-assisted laparoscopy; SPL, single-port laparoscopy.
